# Interplay between SARS-CoV-2 and the type I interferon response

**DOI:** 10.1371/journal.ppat.1008737

**Published:** 2020-07-29

**Authors:** Margarida Sa Ribero, Nolwenn Jouvenet, Marlène Dreux, Sébastien Nisole

**Affiliations:** 1 CIRI, Inserm, U1111, Université Claude Bernard Lyon 1, CNRS, École Normale Supérieure de Lyon, Univ Lyon, Lyon, France; 2 Institut Pasteur, CNRS UMR3569, Paris, France; 3 IRIM, CNRS UMR9004, Université de Montpellier, Montpellier, France; NYU Langone Health, UNITED STATES

## Abstract

The severe acute respiratory syndrome coronavirus-2 (SARS-CoV-2) is responsible for the current COVID-19 pandemic. An unbalanced immune response, characterized by a weak production of type I interferons (IFN-Is) and an exacerbated release of proinflammatory cytokines, contributes to the severe forms of the disease. SARS-CoV-2 is genetically related to SARS-CoV and Middle East respiratory syndrome-related coronavirus (MERS-CoV), which caused outbreaks in 2003 and 2013, respectively. Although IFN treatment gave some encouraging results against SARS-CoV and MERS-CoV in animal models, its potential as a therapeutic against COVID-19 awaits validation. Here, we describe our current knowledge of the complex interplay between SARS-CoV-2 infection and the IFN system, highlighting some of the gaps that need to be filled for a better understanding of the underlying molecular mechanisms. In addition to the conserved IFN evasion strategies that are likely shared with SARS-CoV and MERS-CoV, novel counteraction mechanisms are being discovered in SARS-CoV-2–infected cells. Since the last coronavirus epidemic, we have made considerable progress in understanding the IFN-I response, including its spatiotemporal regulation and the prominent role of plasmacytoid dendritic cells (pDCs), which are the main IFN-I–producing cells. While awaiting the results of the many clinical trials that are evaluating the efficacy of IFN-I alone or in combination with antiviral molecules, we discuss the potential benefits of a well-timed IFN-I treatment and propose strategies to boost pDC-mediated IFN responses during the early stages of viral infection.

## Introduction

**Fig 1:**
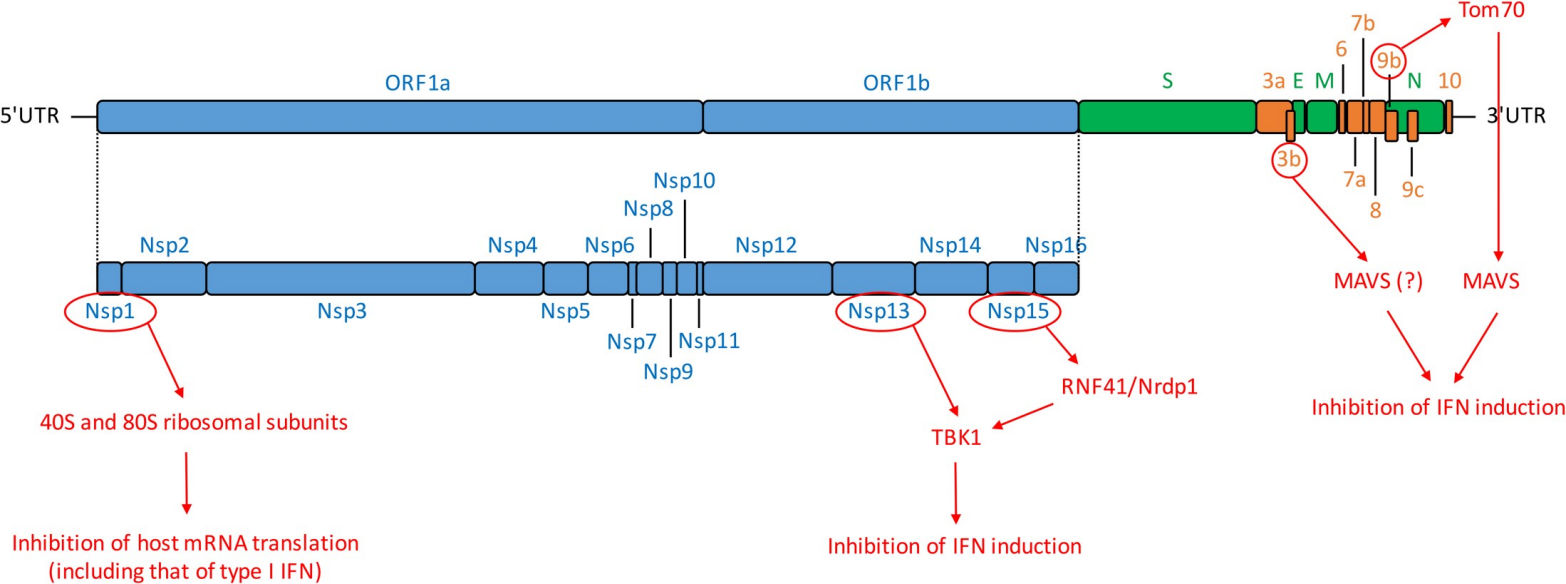
SARS-CoV-2 genomic organization and encoded proteins. ORF1a/1b encode a polyprotein, which is proteolytically processed into Nsp1–16, represented in blue. Structural proteins, including S, E, M, and N proteins are in green. Accessory proteins encoded at the 3′ end of the viral genome comprise ORF3a, 3b, 6, 7a, 7b, 8, 9b, 9c, and 10 and are colored in orange. Untranslated extremities of the genome (5′-UTR and 3′-UTR) are also represented. In red are depicted SARS-CoV-2 proteins that interfere with IFN induction pathway as well as their known or hypothetic target [5, 37, 147]. E, envelope; IFN, interferon; M, membrane; MAVS, mitochondrial antiviral-signaling protein; N, nucleocapsid; Nrdp1, neuregulin receptor degradation protein-1; Nsp, nonstructural protein; ORF, open reading frame; RNF41, ring finger protein 41; S, spike; SARS-CoV-2, severe acute respiratory syndrome coronavirus-2; TANK, TRAF family member-associated NF-κB activator; TBK1, TANK-binding kinase 1; Tom70, translocase of outer mitochondrial membrane 70; UTR, untranslated region.

The severe acute respiratory syndrome coronavirus 2 (SARS-CoV-2) is a beta-coronavirus that emerged at the end of 2019 in China and rapidly spread around the world, causing a pandemic [1, 2]. SARS-CoV-2 infection is responsible for COVID-19, a disease associated with mild symptoms in the majority of cases but that can progress to an acute respiratory distress syndrome [1, 3]. So far (July 16th, 2020), the virus has infected more than 13 million people and caused more than 500,000 deaths worldwide. SARS-CoV-2 is genetically related to other beta-coronaviruses that have caused epidemics: SARS-CoV and MERS-CoV (for Middle East respiratory syndrome-related coronavirus), in 2003 and 2013, respectively. Beta-coronaviruses are enveloped positive-sense single-stranded RNA viruses. The 30-kb genome of SARS-CoV-2 has 82% nucleotide identity with SARS-CoV and contains at least 14 open reading frames (ORFs) [4, 5] (Fig 1). It comprises a 5′-untranslated region (5′-UTR); ORF1a/b, encoding a polyprotein proteolytically processed into 16 nonstructural proteins (Nsp1–16); structural proteins including spike (S), envelope (E), membrane (M), and nucleocapsid (N); 9 accessory proteins (ORF3a, 3b, 6, 7a, 7b, 8, 9b, 9c, and 10); and a 3′-UTR [4, 5] (Fig 1).

Type I interferon (IFN-I) response is critical for providing an efficient protection against viral infections. IFN-I production is rapidly triggered by the recognition by host sensors of pathogen-associated molecular patterns (PAMPs), such as viral nucleic acids [6]. IFN-I–induced signaling converges on transcription factors, which rapidly induces the expression of hundreds of genes called interferon-stimulated genes (ISGs) (reviewed in [7, 8]). This antiviral signaling cascade occurs in virtually all cell types exposed to IFN-I. ISGs, along with other downstream molecules controlled by IFN-I (including proinflammatory cytokines), have diverse functions, ranging from direct inhibition of viral replication to the recruitment and activation of various immune cells [9, 10]. A robust, well-timed, and localized IFN-I response is thus required as a first line of defense against viral infection because it promotes virus clearance, induces tissue repair, and triggers a prolonged adaptive immune response against viruses.

Like most, if not all, RNA viruses, coronavirus RNA is detected by cytosolic sensors including retinoic acid-inducible gene 1 (RIG-I/DExD/H-box helicase 58 [DDX58]) and melanoma differentiation-associated gene 5 (MDA5/IFN induced with helicase C domain 1 [IFIH1]) [11, 12]. Upon activation, RIG-I and MDA5 interact with the downstream adaptor, the mitochondrial antiviral signaling protein (MAVS, also known as IFN-B promoter stimulator 1 [IPS-1], CARD adaptor inducing IFN-beta [CARDIF], or virus-induced signaling adaptor [VISA]) on mitochondria. MAVS activation leads, via the recruitment of tumor necrosis factor receptor-associated factor 3 (TRAF3), TRAF family member-associated NF-_κ_B activator (TANK)-binding kinase 1 (TBK1) and inhibitor of nuclear factor κB (IκB) kinase-ε (IKKε), to the phosphorylation of the IFN gene “master regulators” IFN regulatory factor (IRF)3 and IRF7. Upon phosphorylation, IRF3 and/or IRF7 dimerize and translocate into the nucleus, where they induce the expression of IFN-I and a subset of ISGs referred to as early ISGs (reviewed in [13]). Secreted IFN-I then bind to the interferon alpha and beta receptor (IFNAR, composed of the IFNAR1 and IFNAR2 subunits), leading to the activation of the Jak tyrosine kinases tyrosine kinase 2 (Tyk2) and Janus kinase 1 (JAK1), which in turn phosphorylate the signal transducer and activator of transcription (STAT)1 and STAT2 [14, 15]. Phosphorylated STATs heterodimerize and associate with the DNA binding protein IRF9 to form a complex known as IFN-stimulated growth factor 3 (ISGF3). The ISGF3 complex translocates into the nucleus and binds to interferon-stimulated response elements (ISREs) in ISG promoters, thus inducing the expression of hundreds of ISG products that establish the antiviral state at the site of viral infection [15]. The antiviral response is intensified by various signaling factors, including sensors and transcriptional regulators, which are themselves ISGs induced by ISGF3 and/or directly by the IRF3/IRF7 transcriptional activators. Aside from the IFN-I response, the recognition of double-stranded viral RNA elements by the protein kinase receptor (PKR) triggers a translational arrest in infected cells (reviewed in [8, 16, 17]). This host response is highly connected to the IFN-I response because PKR is also an ISG (reviewed in [16, 18]).

IFN-I response requires fine-tuning because its overactivation is deleterious to the host. Notably, some ISGs are involved in the regulation of cell metabolism, intracellular RNA degradation, translation arrest, and cell death, for which changes can be potentially detrimental to the host. IFN-I also potentiates the recruitment and activation of various immune cells. Thus, although a robust IFN-I response is required as a first line of defense against viral infections, systemic/uncontrolled or prolonged IFN-I production can lead to inflammatory diseases. For example, an exacerbated IFN-I response contributes to the development of autoimmune diseases [19]. COVID-19 is no exception to the rule, and it is therefore critical to understand the regulation of the IFN-I response upon infection.

### SARS-CoV-2 and IFN-I response

SARS-Cov-2 is a poor inducer of IFN-I response in vitro and in animal models as compared with other respiratory RNA viruses [20, 21]. IFN-I levels in the serum of infected patients are below the detection levels of commonly used assays, yet ISG expression is detected [4, 22], thus suggesting that a limited IFN-I production could be sufficient to induce ISGs. Alternatively, IFN-I production could be restricted to specific immune cells, such as plasmacytoid dendritic cells (pDCs). Despite a more efficient replication in human lung tissues, SARS-CoV-2 induced even less IFN-I than SARS-CoV [4], which is itself a weak inducer in human cells [23–25]. An ineffective IFN-I response seems to be a hallmark of other coronavirus infections, as observed with MERS-CoV in ex vivo respiratory tissue cultures [26] and with animal coronaviruses such as the porcine epidemic diarrhea virus (PEDV) or the mouse hepatitis virus (MHV), which are alpha- and beta-coronaviruses, respectively [27, 28]. Indeed, coronaviruses have developed multiple strategies to escape and counteract innate sensing and IFN-I production.

### Inhibition of IFN-I induction and signaling by SARS-CoV-2

**Fig 2:**
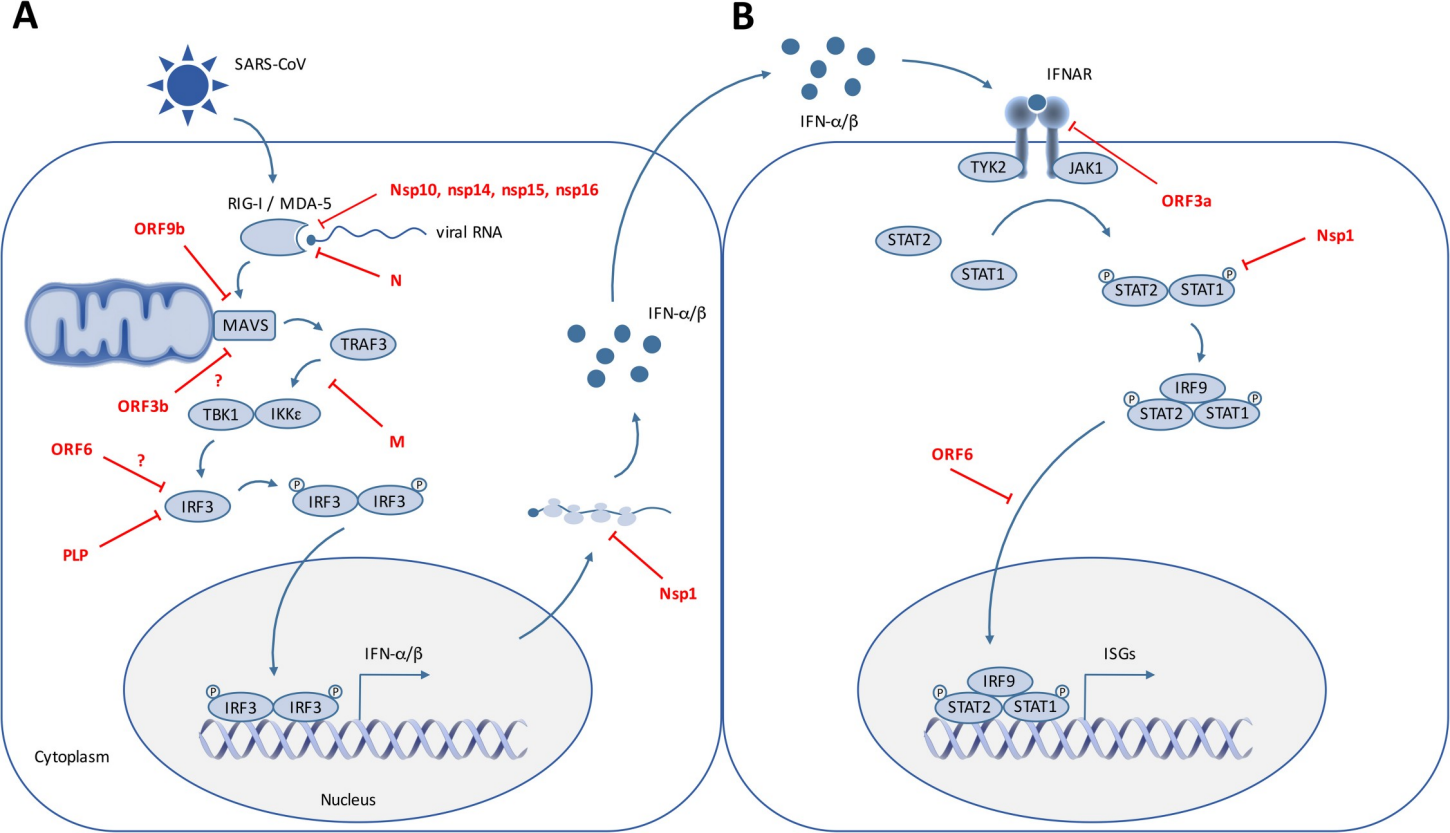
SARS-CoV interfering with IFN induction and signaling. On this cartoon are schematically represented the signaling pathways triggered by SARS-CoV RNA recognition by the cytoplasmic RNA sensors RIG-I and MDA5, which leads to IFN induction (A) and subsequent IFN signaling in surrounding cells, resulting in the expression of ISGs (B). SARS-CoV proteins that have been reported to interfere with these pathways are indicated. IFN, interferon; IFNAR, interferon alpha and beta receptor; IκB, inhibitor of nuclear factor κB; IKKε, IκB kinase-ε; IRF, IFN regulatory factor; ISG, IFN-stimulated gene; JAK, Janus kinase; M, membrane; MAVS, mitochondrial antiviral signaling protein; MDA5, melanoma differentiation-associated gene 5; N, nucleocapsid; Nsp, nonstructural protein; ORF, open reading frame; P, phosphate; PLP, papain-like protease; RIG-I, retinoic acid-inducible gene 1; SARS-CoV, severe acute respiratory syndrome coronavirus; STAT, signal transducer and activator of transcription; TANK, TRAF family member associated NF-κB activator; TBK1, TANK-binding kinase 1; TRAF3, tumor necrosis factor receptor-associated factor 3; TYK2, tyrosine kinase 2.

SARS-CoV encodes at least 10 proteins that allow the virus to either escape or counteract the induction and antiviral action of IFN (Fig 2 and Table 1) [29–48]. Initial observations already suggest that the SARS-CoV-2 anti-IFN arsenal is at least as efficient as that of SARS-CoV [4, 20, 22], although detailed mechanistic studies are required to determine whether the IFN antagonists identified in other coronaviruses have equivalently competent counterparts in SARS-CoV-2. A virus–cell protein interaction map performed with 26 of the 29 SARS-CoV-2 proteins expressed in human embryonic kidney (HEK)293T identified several innate immune signaling proteins as partners of viral proteins cells (Fig 1) [5]. SARS-CoV-2 ORF9b, like SARS-CoV ORF9b, interacts with MAVS through its association with Tom70, thus suggesting a conserved mechanism of IFN-I evasion [5, 40] (Fig 1). Furthermore, SARS-CoV-2 Nsp13 and Nsp15 were found to interact with TBK1 and the TBK1 activator ring finger protein 41 (RNF41)/Nrdp1, respectively [5] (Fig 1). Nsp15, which is a highly conserved endoribonuclease encoded by various coronaviruses, including SARS-CoV [30, 49, 50], antagonizes the induction of IFN-I by cleaving the 5′-polyuridines of the negative-sense viral RNA, as demonstrated for MHV and PEDV in various cellular models [31, 32, 50] (Table 1 and Fig 2). If further validated, the interaction between SARS-CoV-2 nsp15 and TBK1 may reveal that the viral endoribonuclease antagonizes IFN induction via at least 2 mechanisms. SARS-CoV ORF3b was reported to inhibit IFN induction and to act either on IRF3 or possibly on MAVS because it translocates to mitochondria when overexpressed in Vero cells [36, 42]. Despite the fact it encodes a shorter protein than SARS-CoV, SARS-CoV-2 ORF3b was recently found to suppress IFN induction even more efficiently [51]. By screening 15,000 SARS-CoV-2 sequences, the authors identified a natural variant encoding a longer ORF3b and displaying an even greater inhibitory activity [51]. Finally, SARS-CoV-2 Nsp1 was also recently found to bind 40S ribosomal subunits (Fig 1), thus inhibiting host mRNA translation, including that of IFN-I [52], a feature that was previously demonstrated for other coronavirus-encoded Nsp1, including SARS-CoV [43, 45] (Fig 2 and Table 1).

**Table 1:** SARS-CoV proteins interfering with IFN-I induction and signaling.

Protein	Mechanism	Affected Step	Experimental Approach	Cellular Model	References
**Inhibition of IFN-I Induction**					
Nsp14	Guanine-N7-methyltransferase activity—methylates capped RNA transcripts	Sensing	Yeast genetic system—validated using a SARS-CoV replicon	Yeast	[29]
Nsp15 (EndoU)	Cleaves the 5′-polyuridines from negative-sense viral RNA	Sensing	Protein overexpression (mutants for other coronaviruses)	MA104 cells	[30–32]
Nsp16	2′-O-methyltransferase activity involved in viral RNA capping	Sensing	SARS-CoV mutants	Vero, Calu-3, and HAE cells; mice	[33]
Nsp10	Cofactor of Nsp16, required for RNA cap methylation	Sensing	In vitro reconstitution of SARS-CoV mRNA cap methylation	In vitro	[34]
N	Inhibits TRIM25-mediated RIG-I ubiquitination	Sensing	Protein overexpression	HEK293T and A549 cells	[35–37]
Nsp3 (PLP)	Deubiquitinates cellular substrates, possibly including RIG-I	Sensing	Protein overexpression	HEK293 cells	[38]
	Inhibits IRF3 phosphorylation	IRF3 activation	Protein overexpression	HEK293, HeLa, and MA104 cells	[30, 39]
ORF9b	Targets MAVS, TRAF3, and TRAF6 to degradation	Downstream signaling	Protein overexpression	HEK293 and A549 cells	[40]
M	Impedes the formation of TRAF3/TBK1/IKKε complex	Downstream signaling	Protein overexpression; also observed in infected cells	HEK293 and HeLa cells	[41]
ORF6	Unknown	Downstream signaling	Protein overexpression	HEK293T and A549 cells	[36]
ORF3b	Mechanism unclear; may target MAVS	Downstream signaling	Protein overexpression	HEK293T and A549 cells	[36, 42]
Nsp1	Promotes cellular mRNA degradation and blocks host mRNA translation	Expression of IFN-I	Protein overexpression—validated using a SARS-CoV mutant virus	HEK293 and Vero cells	[43–45]
**Inhibition of IFN-I Signaling**					
ORF3a	Promotes IFNAR1 degradation	IFN binding to its receptor	Protein overexpression	Huh7 cells	[46]
Nsp1	Decreases STAT1 phosphorylation	STAT1 activation	Protein overexpression—validated using a SARS-CoV mutant virus	HEK293T and Vero cells	[47]
ORF6	Sequesters karyopherin alpha 2 and beta 1	Nuclear translocation of STAT1	Protein overexpression—Validated using a SARS-CoV mutant virus	HEK293(T), A549, and Vero cells	[36, 48]

**Abbreviations:** EndoU, endoribonuclease; HAE, human airway epithelial; HEK, human embryonic kidney; IFN, interferon; IFNAR, interferon alpha and beta receptor; IFN-I, type I IFN; IκB, inhibitor of nuclear factor κB; IKKε, IκB kinase-ε; IRF, IFN regulatory factor; M, membrane; MAVS, mitochondrial antiviral signaling protein; Nsp, nonstructural protein; ORF, open reading frame; PLP, papain-like protease; RIG-I, retinoic acid-inducible gene 1; SARS-CoV, severe acute respiratory syndrome coronavirus; STAT, signal transducer and activator of transcription; TANK, TRAF family member-associated NF-κB activator; TBK1, TANK-binding kinase 1; TRAF, tumor necrosis factor receptor-associated factor; TRIM25, tripartite motif containing 25.

Another viral strategy to inhibit IFN-I signaling is to enhance the host retrocontrol of this pathway. Several ISGs are themselves repressors of the IFN-I response, and their regulatory functions operate at the viral and host mRNA transcription and translation steps, acting via a wide-range of mechanisms (reviewed in [7, 53]). For example, the inducible negative regulators such as the suppressor of cytokine signaling (SOCS1 and SOCS3) act at various levels of the Jak–STAT pathway or by targeting IRF7 for degradation [54]. In the context of SARS-CoV, the S protein induces the expression of SOCS3 expression in B cells [55]. Induction of SOCS1/3 expression is also detected in SARS-CoV–infected cells, albeit to a lower extent as compared with other respiratory viruses [56]. Recent genomic screen approaches identified a set of repressors of the IFN-I response depending on the cell type and activation pathway involved [57–59]. Hence, one might anticipate that distinct repressors of the IFN-I response are induced depending on the cell type targeted by SARS-CoV-2, the level of replication, and the microenvironment. For example, in the context of coronaviruses, an inefficient detection of MHV infection likely results from an inhibition of the basal levels of sensors mRNA expression in several cell types [60]. It is conceivable that this inhibition might involve negative regulators such as the IFN-inducible RNF125, which targets signaling components such as RIG-I, MDA5, and MAVS for degradation [61].

### Interplay between host translation and the IFN-I response

Inhibition of protein synthesis is a conserved host response to prevent viral infections. The host translation is dynamically regulated by PKR, activated via recognition of viral RNA (reviewed by [62]). Activated PKR inhibits the eukaryotic initiation factor 2 (eIF2α), a major regulator of the initiation phase of mRNA translation, by phosphorylating its α subunit. The PKR-induced translational arrest shuts down the negative feedback on the IFN-I response, which can thus result in a prolonged and/or amplified IFN-I response [63]. Because PKR is an ISG, the translational arrest is, in turn, potentiated by the IFN-I response (reviewed in [64]). This highlights a paradoxical situation in which translation arrest prevents viral replication but also set a threshold of viral detection to commensurate the host transcriptional antiviral response to the level of infection [63].

Whether the PKR pathway is modulated by SARS-CoV2 is unknown, yet different coronaviruses regulate PKR-eIF2α axis and host translation. For example, the MERS-CoV protein 4a (p4a) accessory protein impedes PKR activation [65, 66]. Future studies are needed to further uncover the relationship between IFN-I response and host translation and their dynamics in the context of SARS-CoV2 infection.

## Dynamics of the IFN-I response define severity of infection

### Dynamics of the IFN-I response at the levels of individual cells and cell population

The IFN-I response varies among different cell types and within different microenvironments. Studies at the single-cell level suggest that the amplitude and kinetic of the response is also heterogeneous for a given cell type. Mathematic modeling revealed that IFN-I response is, at least in part, stochastic because only a fraction of cells are able to produce IFN-I upon activation by agonists of the sensors and are sensitive to the paracrine stimulation by IFN-I [67–71]. The heterogeneity of the IFN-I response can be imprinted by the state of the cell at the activation time, including its global translation activity, metabolism, expression levels of signaling molecules (sensors, adaptors, and receptors) [67–70]. Additionally, the distinct onsets of the IFN-I induction depend on the rapidity and amplitude of viral replication. This heterogeneous responsiveness at the individual cell level consequently shapes the dynamics of the host antiviral response at the whole population level [69–71]. This model of the IFN-I response dynamics yielded in the context of various RNA viruses provides a framework likely at play for coronavirus infections. A delayed induction of the ISG expression via virus-induced modulation of the basal activity of transcriptional activity of STAT1 and PKR pathways leads to a peak of coronavirus replication preceding the ISG response [72]. Additionally, in vivo study of the dynamic of MHV infection showed that a fast and robust IFN-I production by pDCs down-regulate the IFN-I response by other cells [73]. This suggests that the IFN-I response at play in different cell types might drive the control of coronavirus infection and potentially contribute to the progression of the disease.

### Impaired or delayed IFN-I response contributes to the disease

**Fig 3:**
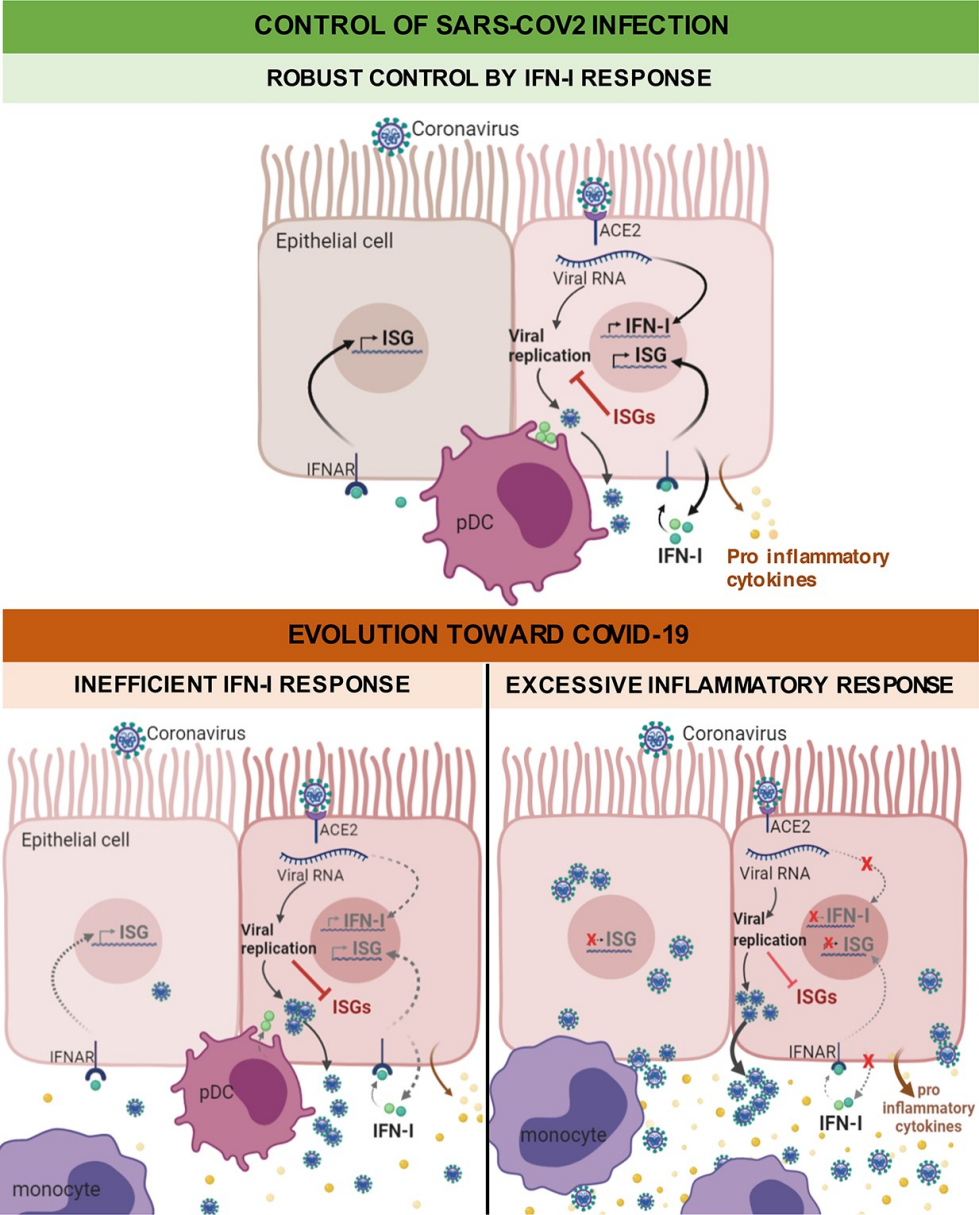
Working model of the failure of IFN-I to control of SARS-CoV-2 infection, leading to COVID-19. While IFN-I can control viral infection (upper panel), IFN-I deficiency is believed to play a key role in SARS-CoV-2 pathogenesis (lower panel). As shown for related coronaviruses, a delayed IFN-I signaling is associated with robust virus replication and severe complications, i.e., inflammation and “cytokine storm,” notably via the accumulation of monocytes resulting in lung immunopathology, vascular leakage, and suboptimal T cell response. ACE2, angiotensin I converting enzyme 2; IFNAR, interferon alpha and beta receptor; IFN-I, type I interferon; ISG, IFN-stimulated gene; pDC, plasmacytoid dendritic cell; SARS-CoV-2, severe acute respiratory syndrome coronavirus-2.

As mentioned above, coronaviruses possess various mechanisms to defeat the IFN-I response within infected cells, and this inhibition ability is associated with clinical severity (reviewed in [74]). Clinical studies showed that coronaviruses evade innate immunity during the first 10 days of infection, which corresponds to a period of widespread inflammation and steadily increasing viral load [75, 76]. Elevated virus replication eventually leads to inflammation and hypercytokinemia, referred to as a “cytokine storm” [77–80] (Fig 3). The delayed IFN-I response indeed promotes the accumulation of pathogenic monocyte–macrophages [77, 81]. This cell infiltrate results in lung immunopathology, vascular leakage, and suboptimal T cell response [77, 81]. Immune phenotypic profiling in peripheral blood mononuclear cells (PBMCs) of COVID-19 patients similarly revealed that high viremia is associated with an exacerbated IFN-I response, an aggravated cytokine secretion, and inflammation, driving clinical severity [22]. Although the IFNAR signaling pathway was up-regulated at an earlier disease stage, down-regulation of ISGs, together with exacerbated NF-κB activation, promotes a cytokine storm and hyperinflammation, found in critically ill patients [22].

Collectively, these findings highlight the negative impact of a delayed IFN-I response on viral control and disease severity. However, the underlining mechanisms that drive the temporal control of the IFN-I response in patients are still elusive. In particular, the host and viral determinants driving the on/off switch of the IFN-I response in infected cells, noninfected cells, and/or stimulated immune cells need to be investigated. Such studies will certainly benefit from longitudinal studies of immune profiling in SARS-CoV2 infected patients at the single-cell level and in combination with the clinical data.

### Robust and localized IFN-I response by pDCs

By producing 1,000-fold more IFN-I than any other cell types, pDCs are at the heart of the antiviral IFN-I response [82, 83]. They also produce other proinflammatory cytokines, which contribute to modulate the function of several immune cells, such as the mobilization of natural killer (NK) cells or the licensing of virus-specific T cell responses [82–85]. Because pDCs are refractory to most viral replication, their antiviral response cannot be inhibited by viral proteins [82, 86]. This unopposed response likely contributes to the exceptional magnitude of pDC IFN-I production [82, 87–89]. pDCs are circulating immune cells; nonetheless, their response is mostly localized at the infection site because their activation requires physical contact with infected cells [82, 83, 90]. The contact site between pDCs and infected cells, which we named the interferogenic synapse, is a specialized platform for PAMP transfer from infected cell to the Toll-like receptor 7 (TLR7) sensor in pDC, leading to an antiviral response [91].

Previous studies on SARS- and MERS-CoV demonstrated that the rapid production of IFN-I by pDCs is essential for the control of potentially lethal coronavirus infections in mouse models [77, 92]. pDCs migrate into the lungs at the early phase of infection (i.e., pDC number peaks at day 2), temporally coinciding with the peak of IFNα production [92, 93]. The pDC number was found to be reduced in blood of COVID-19 patients as compared with control patients [22], potentially resulting from a prior response followed by a vanished number of circulating pDCs and/or their mobilization to the infected site. Future studies are needed to address how pDC responsiveness evolves in the course of SARS-CoV-2 infection and how pDCs respond to contact with coronavirus-infected cells.

## Are IFNs protective of or detrimental to SARS-CoV-2?

Despite the abovementioned viral inhibitory mechanisms of IFN-I response (Table 1), exogenous IFN-I in cell cultures efficiently inhibit SARS-CoV, SARS-CoV-2, and MERS-CoV spread [5, 20, 26, 51, 94–101]. Consistently, IFN-I was shown to have a protective effect against SARS-CoV and MERS-CoV, alone or in combination with other antivirals, in various animal models including mice, marmosets, and macaques [97, 102, 103]. IFN-I and III interfere with virus infection by inducing the expression of several hundred ISGs [7]. Numerous well-described ISGs exhibit direct antiviral activities by targeting specific stages of the viral life cycle, including entry into host cells, replication, protein translation, and assembly of new virus particles. As mentioned above, many signaling regulators are themselves ISGs, thus leading to amplification of the antiviral IFN-I pathway.

### Detrimental effects of IFNs on SARS-CoV-2 replication

As a first step towards identifying ISGs able to restrict SARS-CoV-2 replication, transcriptomic responses to infection have been analyzed in different cellular models, including primary cells, organoids, and clinical samples [20, 104–106], as summarized in Table 2. These studies demonstrate that, despite triggering very little to no IFN expression (Table 2), SARS-CoV-2 replication induces moderate levels of a limited number of ISGs. A small subset of infected cells may be refractory to the antagonistic mechanisms of SARS-CoV-2, producing minute but sufficient amounts of IFNs to trigger ISG induction in larger population of cells. Alternatively, ISGs may be up-regulated in noninfected cells, which were analyzed together with infected ones. Indeed, interpretation of genome-wide investigations of virus–pathogen interactions are often obscured by analyses of mixed populations of infected and uninfected cells [107].

**Table 2:** ISGs and IFN signature of SARS-CoV-2–infected samples.

Infection (Viral Dose and Time Point)	Cellular Model	ISGs and IFN Signatures (Top Up-Regulated ISGs)	References
MOI 0.2 24 h	A549 lung alveolar cells expressing ACE2	Moderate up-regulation of a subset of ISGs; no IFN-I/III	[20]
MOI 2 24 h	A549 lung alveolar cells expressing ACE2	High induction of ISGs (OASL, IFIT1, IFIT2, IFIT3, OAS2) and IFNs	[20]
MOI 2 24 h	Lung Calu-3 cells	High induction of ISGs (TRIM22, MX2, IFIT2, IFIT3, and RSAD2/viperin) and IFNs	[20]
MOI 2 24 h	NHBE cells	A small subset of ISGs induced (IFI27, MX1, OAS1, MX2, and IFITM1); undetectable level of IFN	[20]
N/A	Postmortem lung samples from 2 COVID-19 patients	A subset of ISGs (IFI6, IFIT1, OAS1, IFITM2, and IFIT3); undetectable level of IFN-I/III	[20]
N/A	BALF of 8 COVID-19 patients	IFIT family members and IFITM family members, as well as ISG15 and RSAD2/viperin	[105]
MOI 1 60 h	Expending human intestinal organoids	Broader response than in lung cells, ISGs (IFI6, IFI27, IFITM3, MX1, and RN7SK); low expression of IFN-I/III	[106]

**Abbreviations:** ACE2, angiotensin I converting enzyme 2; BALF, bronchoalveolar lavage fluid; IFI, interferon-inducible protein; IFIT, interferon-induced protein with tetratricopeptide repeats; IFN, interferon; IFN-I/III, type I/III IFN; ISG, IFN-stimulated gene; MOI, multiplicity of infection; MX, myxovirus resistance protein; N/A, not applicable; NHBE, normal human bronchial epithelial; OAS, 2′-5′-oligoadenylate synthetase; RSAD2, Radical S-Adenosyl Methionine Domain Containing 2; RN7SK, RNA component of 7SK nuclear ribonucleoprotein; SARS-CoV-2, severe acute respiratory syndrome coronavirus-2; TRIM22, tripartite motif containing 22.

Of note, by contrast to low-multiplicity of infection (MOI) infection of A549 cells expressing angiotensin I converting enzyme 2 (ACE2), normal human bronchial epithelial (NHBE) cells, and patient samples, high-MOI infections of A549-ACE2 and Calu-3 cells led to the high induction of IFNs and ISGs, including ISGs with broad antiviral activities [20] (Table 2). This discrepancy of IFN production/signaling between the levels of viral replication and/or proportion of infected cells might reflect that the counteraction measures employed by SARS-CoV-2 are less potent at high MOI. Alternatively, as suggested by Blanco-Melo and colleagues, high-MOI infections in cell culture may generate more PAMPs, such as defective noninfectious viral particles, than low-MOI infections [108].

Despite being expressed at moderate levels in vitro and in vivo, several up-regulated ISGs identified by these transcriptomic studies (Table 2) exhibit well-characterized broad-spectrum antiviral activities and could thus have additive restrictive effects on SARS-CoV-2 replication. For instance, the 3 members of the interferon-induced protein with tetratricopeptide repeats (IFITM) family, known to inhibit entry of numerous enveloped RNA viruses [109], similarly restrict entry of SARS-CoV, MERS-CoV, and the globally circulating human coronaviruses 229E and NL63 in 293T and A549 cell lines [110, 111]. OAS1 and mycovirus resistance protein (Mx)A could also contribute to the IFN-I–mediated inhibitory effect on SARS-CoV-2 because a clinical study revealed that single nucleic polymorphisms in the OAS1 3′-UTR and MxA promoter region appear associated with host susceptibility to SARS-CoV in the Chinese Han population [112]. Moreover, the fact that MERS-CoV nonstructural protein 4B (NS4b) is a 2′-5′-oligoadenylate synthetase (OAS)-RNase L antagonist [113] suggests that the OAS pathway contributes to the antiviral effects of IFNs on coronavirus replication. ISGs positively potentiating IFN signaling, such as IFIH1/MDA5, TANK, IRF7, and STAT1, were also increased in the bronchoalveolar lavage fluid (BALF) of COVID-19 patients as compared with healthy controls [105] and could potentially contribute to the amplification of IFN-I response against SARS-CoV-2 replication.

Zinc finger antiviral protein (ZAP), which is encoded by an ISG, contributes to the anti-SARS-CoV-2 effect of IFNs in human lung Calu-3 cells [114]. ZAP is known for restricting the replication of numerous viruses such as retroviruses and filoviruses [115]. The protein recruits the cellular mRNA degradation machinery to viral RNA via 5′-C-phosphate-G-3′ (CpG) dinucleotide recognition [115].

To further determine which individual ISG or combination of ISGs mainly restricts SARS-CoV-2 replication in vitro, several previously established approaches could be used, such as, for example, screening for single or combined ISG activity using a lentiviral vector-based library, as successfully performed by Schoggins and colleagues for other viral infections [116–119]. Indeed, this library of around 380 human ISGs was recently screened in human hepatoma cells for antiviral activity against HCoV-229E [120]. The screen identified IFN-inducible lymphocyte antigen 6 complex, locus E (LY6E) as a potent inhibitor of the replication of multiple coronaviruses, including SARS-CoV, SARS-CoV-2, and MERS-CoV, by blocking fusion of viral and cellular membranes [120]. Mice studies revealed that LY6E directly protects primary B cells and dendritic cells from murine coronavirus infection [120]. Pursuing the identification and characterization of IFN effectors with potent anti-SARS-CoV-2 activities will reveal weakness points in the life cycle of SARS-CoV-2 and may lead to the design of drugs that activate antiviral ISGs or either mimic or amplify their action.

### Beneficial effects of IFN on SARS-CoV-2 replication

Recent advances in systematic screening strategies have revealed the existence of a small subset of ISGs exhibiting proviral activities [116, 119]. These proviral ISGs act either by exhibiting direct proviral activities such as facilitating viral entry [119] or via their abilities to negatively regulate IFN signaling and facilitate the return to cellular homeostasis. The receptor tyrosine kinase AXL is a well-characterized example of an ISG that is used by enveloped virus for cellular internalization [121–123]. Alternatively, ISGs that possess antiviral activities against a viral family can be hijacked by unrelated viruses to favor infection. This is the case for IFITM2 and IFITM3, which potently block entry of a broad range of enveloped viruses [109] while promoting entry step of human coronavirus OC43 (HCoV-OC43) in human cells [124].

SARS-CoV-2 uses ACE2 and transmembrane serine protease 2 (TMPRSS2) to enter cells [125]. Viral tropism is thus largely dictated by ACE2 and TMPRSS2 coexpression. Analysis of human, nonhuman primate, and mouse single-cell RNA-sequencing (scRNA-seq) data sets generated from healthy or diseased individuals revealed that expression of *ACE2* is primarily restricted to type II pneumocytes in the lung, absorptive enterocytes within the gut, and goblet secretory cells of the nasal mucosa [126]. Interestingly, this meta-analysis identified an association between ACE2 expression and canonical ISGs or components of the IFN-signaling pathway in different tissues. Independent analyzes of publicly available data sets concluded that ACE2 expression pattern is similar to ISGs [127]. In vitro validations were performed by treating primary human upper airway cells with numerous inflammatory cytokines. IFNα2, and to some extent IFNy, led to greater and more significant ACE2 up-regulation compared with all other tested cytokines [126]. Substantial up-regulation of *ACE2* was also observed in primary skin and primary bronchial cells treated with either IFN-I or IFN-II. Moreover, ACE2 expression was also up-regulated upon ex vivo influenza A infection in human lung explants isolated following surgical resection [126]. Because the majority of cells robustly up-regulating ACE2 were epithelial, this observation potentially explains why previous analyses to define canonical ISGs within immune populations did not identify *ACE2* as an induced gene [116]. Finally, STAT1, STAT3, IRF8, and IRF1 binding sites were identified within −1,500 to +500 bp of the transcription start site of ACE2 [126]. Despite need for direct evidence that IFNs up-regulate ACE2 in target cells in vivo, altogether these studies suggest that ACE2 could be an ISG that enhances SARS-CoV-2 internalization in human epithelial cells [125, 126].

Elucidating tissue and cell type specificity of ISGs, as well as their mechanisms of action, is essential for understanding the potential dual role of IFNs during human SARS-CoV-2 infection. It may also guide the use of IFNs in clinical trials.

### Clinical implications of the dual role of IFN on SARS-CoV-2 replication

Although IFN-I treatment gave some encouraging results against SARS-CoV and MERS-CoV in vitro and in animal models, including mice, marmosets, and macaques [97, 102, 103, 128, 129], additional knowledge to optimize its therapeutic efficiency in humans is required [130–134]. Previous information yielded from these animal studies provided guidance for treating the current pandemic virus. First, it became clear from these former studies that IFNβ is a more potent inhibitor than IFNα as shown both in vitro and in patients [129, 132]. Second, the timing of IFN-I treatment seems determinant for infection outcomes. Indeed, as shown in mice and in macaques, IFN-I is protective when administered prior to SARS-CoV or MERS-CoV infection or early in the course of infection, whereas late administration could be either ineffective or detrimental [77, 135]. In humans as well, IFN-I–based therapies were not beneficial to critically ill patients with multiple comorbidities and who were diagnosed late with MERS-CoV, thus pointing out that IFN-I has to be administered early after infection [134, 136].

The first clinical trials using IFN-I alone or in combination with other antivirals are currently carried out in COVID-19 patients in several countries. For instance, the multicenter, adaptive, randomized, open clinical trial DisCoVeRy evaluates, among other treatment, the efficacy of IFNβ as a treatment for COVID-19 in hospitalized adults in Europe. A recent open-label, randomized, phase 2 trial performed in adults with COVID-19 in Hong Kong showed that the triple combination of IFNβ-1b, lopinavir–ritonavir, and ribavirin was safe and superior to lopinavir–ritonavir alone in alleviating symptoms and shortening the duration of viral shedding and hospital stay in patients with mild to moderate COVID-19 [137]. It has to be noted that the patients were treated in the early stages of the disease because the median number of days from symptom onset to start of study treatment was 5 days, further reinforcing the fact that the timing of IFN-I treatment is key [137].

Other therapeutic approaches are under investigation to avoid the adverse effects of IFN-I therapy and/or its potential inefficacity when administrated too late postinfection. One strategy is to use aerosol formulations of recombinant IFN to deliver the cytokine directly inside the lung [138, 139]. This approach has several benefits because it is a noninvasive route of administration, and the local concentration reached in the tissue can be higher than through systemic injection and is thus expected to minimize the adverse effects of IFN. Nebulized IFNα-2b was used on COVID-19 patients in Wuhan, alone or in combination with arbidol [140]. The study, performed on 77 adults, showed a significant reduction of the duration of detectable virus in the upper respiratory tract in IFNα-2b–treated patients, with or without arbidol [140]. Another study currently ongoing in Beijing aims at evaluating the efficacy and safety of recombinant human IFNα spray in preventing SARS-CoV-2 infection in highly exposed medical staffs (ChiCTR2000030013).

Type III IFNs (IFNλs or IFN-III) are gaining an increased interest in antiviral therapies [141–143]. Like IFN-I, they activate the JAK–STAT signaling pathway. They do so via a receptor that is largely restricted to cells of epithelial origin, including respiratory epithelial cells (reviewed in [144]). IFN-IIIs are induced upon viral infections, and they are growing evidence that they provide important first-line defense against viral infections of the respiratory and gastrointestinal tracts [145–147]. In mice, IFN-III was shown to protect epithelial cells of the respiratory and tract from infections with several respiratory viruses, including MERS-CoV [147]. A study investigating SARS-CoV-2 infection of intestinal epithelial cells, using both colon-derived cell lines and primary colon organoids, showed that IFN-III response was more efficient than IFN-I at controlling viral replication [148]. However, IFN-IIIs produced by dendritic cells in the lung were recently shown to cause barrier damage and to compromise host tissue tolerance and predispose to lethal bacterial superinfections [149]. Therefore, although the antiviral properties are promising, the benefit of IFN-III to treat COVID-19 patients awaits careful evaluation. The first clinical trials using IFN-III are ongoing, including one launched at the Massachusetts General Hospital to evaluate the safety and efficacy of pegylated IFNλ on a small number of COVID-19 patients (NCT04343976).

Besides the use of recombinant IFN as a therapeutic treatment, one interesting alternative strategy would be to boost the natural innate immune defenses of COVD-19 patients at early stages of the disease. Because pDCs are seemingly crucial to control coronavirus infections [77, 92], a possibility would be to either amplify or prolong their activation to make them produce more IFN-I and IFN-III. A number of negative feedback loops prevent an exacerbated activation of pDCs, which can be deleterious for the organism in the long term. Thus, transitorily inhibiting these negative retrocontrols may increase the antiviral activity of pDCs. For instance, the bone marrow stromal cell antigen 2 (BST2) is an ISG that activates the immunoglobulin-like transcript 7 (ILT7) inhibitory receptor expressed by pDCs to interrupt the IFN-I response [150]. The blockade of this interaction using either antibodies or inhibitory molecules should thus increase the duration of pDC activation. One could also envisage to take advantage of viral proteins that counteract the antiviral activity of BST2, such as HIV-1 viral protein U (vpu) [151]. Other pDC inhibitory molecules include natural monamines such as histamine, dopamine, or serotonin, which bind to the C-X-C motif chemokine receptor 4 (CXCR4) at the surface of pDCs [152]. Because the CXCR4 antagonist AMD3100 (also known as plerixafor) blocks the binding of monoamines to pDCs, it can prevent the amine-dependent inhibition of pDC activation [152]. AMD3100 is already used in clinics as an immunostimulatory molecule able to mobilize hematopoietic stem cells in cancer patients [153]. Finally, we recently reported that the peptidyl-prolyl isomerase peptidyl-prolyl *cis*-*trans* isomerase NIMA-interacting 1 (Pin1) switches off the IFN-I expression by pDCs by inducing IRF7 degradation [154]. A number of Pin1 inhibitors have been developed and could be tested for their potential activity on human pDCs [155] and could represent another possible therapeutic strategy to boost pDC-mediated IFN-I production.

## Conclusions and perspectives

SARS-CoV-2 emerged in the human population around 7 months ago, yet it seems well adapted to avoid and inhibit the IFN-I response in its new host. Such efficient strategies allow the virus to replicate and disseminate in infected individuals without encountering the initial host defense. This modest IFN response could explain why viremia peaks at early stages of the disease, at the time of symptoms appearance, and not around 7 to 10 days following symptoms, like during SARS-CoV and MERS-CoV infections. IFNβ treatment would be expected to improve the antiviral response of patients at the early stage of COVID-19 and, if possible, at the site of infection. Indeed, IFNβ appeared to be pivotal to improve patient states in a combined therapy regiment of IFNβ, lopinavir–ritonavir, and ribavirin [137]. Nonetheless, IFN-resistant viral mutants may arise and be able to control IFN even more efficiently than parental viruses.

The exacerbated production of proinflammatory cytokines observed at later stage of COVID-19 might challenge the efficiency of an IFNβ treatment administrated after appearance of symptoms. There is indeed an increasing appreciation of the detrimental effects of inappropriate, excessive, or mistimed IFN-I responses in viral infections [156]. The underlying mechanisms by which IFN-I promote disease severity likely include immunopathology due to excessive inflammation and direct tissue damage. At the late stages of COVID-19, immunomodulatory drugs that diminish inflammation may benefit patients. The therapeutic benefits of such treatments have been demonstrated in the context of influenza infection in clinical trials [157] and in mice models [158]. Discovery of host markers associated with disease progression will be instrumental to defining the appropriate treatment and time of administration.

Luckily, most COVID-19 patients develop no or mild symptoms. In these patients, the virus ends up being cleared by the immune system, possibly through a partial protection conferred by cross-reactive CD4+ T cells that have been found in between 40% and 60% of unexposed individuals [159]. It is therefore probable that a viral replication that is under control thanks to an efficient adaptive immunity prevents a systemic viral spread and the subsequent cytokine storm. Prior and coinfections along with age, gender, immunological state, and comorbidities also likely play a key role in the ability of the patients to efficiently respond to SARS-CoV-2 infection.

The current studies that aim to better understand the mechanisms that render some patients particularly sensitive to SARS-CoV-2 infections raise hope for the possibility of treating patients with drugs that either enhance the IFN response at the early stage of the disease or dampen it at later stages.
